# The Effects of Cannabis Access Laws on Sleep in the U.S.

**DOI:** 10.1016/j.focus.2025.100387

**Published:** 2025-07-04

**Authors:** Carol Xu, Zachary Sturman

**Affiliations:** O’Donnell School of Public Health, University of Texas Southwestern Medical Center, Dallas, Texas

**Keywords:** Health economics, cannabis, sleep, policy

## Abstract

**Introduction:**

The global average sleep duration has declined significantly, with substantial health and economic consequences. Given the increasing legalization of cannabis for medical and recreational use, this study examines the association between cannabis access laws and sleep duration in the U.S.

**Methods:**

A two-way-fixed-effects difference-in-differences regression was used to analyze the impact of medical and recreational cannabis laws on sleep duration. Data were drawn from the American Time Use Survey, including 175,493 respondents aged 18–65 years from 2003 to 2021. Subgroup analyses were performed on sex and age groups. Analyses were conducted in 2024.

**Results:**

Recreational cannabis laws were associated with an average reduction in sleep of 5.37 minutes per night (99% CI=0.91, 9.83), whereas medical cannabis laws had no significant effect on sleep duration. Recreational cannabis laws were associated with individuals falling asleep 7.14 minutes later (99% CI=3.12, 11.16) with no change in wake-up times, and only the eating and drinking category of time use was significantly affected, with an increase of 1.90 minutes. These findings were concentrated in males and those aged ≥25 years.

**Conclusions:**

The implementation of recreational cannabis laws has a negative effect on sleep duration, highlighting the need for further research on the broad impacts of cannabis access on health.

## HIGHLIGHTS

• Recreational cannabis access laws are associated with later sleep onset.
• Sleep duration was 5.37 minutes shorter after recreational legalization.
• Medical cannabis laws are not associated with sleep duration.
• Strongest sleep changes for males and adults aged ≥25 years.
• Recreational laws are linked to more time spent eating and drinking.

## INTRODUCTION

The global average duration of sleep has declined from 8 hours per night in 1942 to just 6.8 hours today. Health officials recommend at least 7 hours of sleep per night, yet only 40% of Americans achieve this amount.^1^ A meta-analysis concluded that short sleepers—those getting <7 hours per night of sleep—have a 12% increased mortality risk as well as a heightened likelihood of other physical ailments.^2^ This mortality risk increase is the equivalent of that caused by drinking 5–6 alcoholic beverages per day.^3^ Economists also recognize the broader societal impacts of poor sleep, including decreases in earnings^4^; lowered performance on standardized tests^5^; poorer indicators of mental well-being^6^; increases in fatal automobile accidents^7^; and elevated risks of obesity, diabetes, cardiovascular disease, and breast cancer.^8^ The rapid decline in average sleep as well as documented relationships between sleep and various health measures—including hypertension, poor cognitive functioning, memory problems, mood disorders, cardiovascular disease, Type 2 diabetes, and various cancers—prompted the U.S. Centers for Disease Control in 2014 to declare inadequate sleep a public health epidemic.^9^

Amid this growing concern, the legalization of cannabis for medical and recreational use has raised questions about its potential influence on sleep health. As of 2021, cannabis is legal for recreational use in 19 states and for medical use in 37. Although reported prevalence rates vary, cannabis is commonly used as a sleep therapy, with one study suggesting up to 80% of cannabis users using it for this purpose.^10^ Evidence also indicates that individuals may substitute over-the-counter sleep aids with cannabis when it becomes legally available.^11^ However, the effects of cannabis on sleep duration and quality remain unclear. Existing literature suggests that although cannabis might help people fall asleep sooner, it may also worsen overall sleep quality and has uncertain associations with sleep duration.^12^

This paper seeks to examine the association between cannabis access laws and sleep. Whereas clinical studies may rely on small, nonrepresentative trials or associational comparisons between cannabis users and nonusers, this paper exploits the variation in cannabis access law timing to offer clearer insights into how legal access to cannabis impacts sleep patterns. This strategy has been implemented in previous economics literature, which has found that medical cannabis laws lead to a decrease in obesity risks,^13^ a decrease in college students’ time spent on education-related activities,^14^ and an increase in sexual activity.^15^ Evidence from this study has important implications for policymakers, healthcare providers, and consumers because understanding the relationship between cannabis access and sleep will influence public health strategies, healthcare recommendations, and the regulation of cannabis products.

## METHODS

### Study Sample

**Figure 1:**
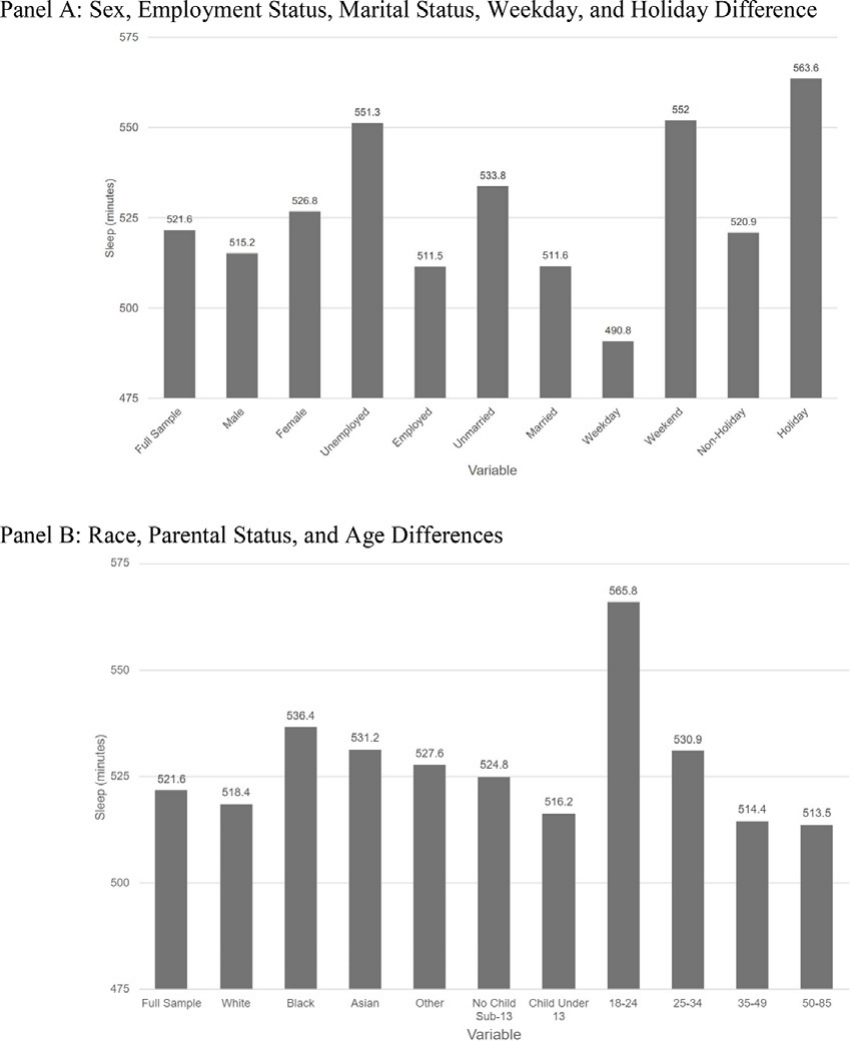
Mean nightly sleep duration by subgroup in the American Time Use Survey. (A) Sex, employment status, marital status, weekday, and holiday difference. (B) Race, parental status, and age differences.

The American Time Use Survey (ATUS)^16^ is a nationally comprehensive survey conducted by the Bureau of Labor Statistics to measure how people in the U.S. allocate their time across various activities, including sleep, work, household chores, childcare, and leisure. The ATUS respondents are selected from households that have participated in the Current Population Survey, allowing survey responses to be linked to demographic information. Owing to the oversampling of more sparsely populated states, survey weights were included to produce nationally representative estimates. Table 1 shows basic demographic information for ATUS respondents in comparison with the U.S. general population, and Figure 1 provides basic descriptive statistics on sleep duration for the sample used in this study. ATUS data were obtained for all 50 states and the District of Columbia from 2003 to 2021. All respondents aged between 18 and 65 years were eligible for this study, resulting in a total sample size of 175,493 respondents. Survey responses included average sleep duration, falling asleep and waking up times, age, sex, race, parental status, employment status, and marital status.

**Table 1:** ATUS Demographics, Summary Statistics

Covariate	ATUS sample (share)	U.S. population (share)
Male	0.45	0.49
Married	0.55	0.54
White	0.80	0.62
Employed	0.75	0.62
Veteran	0.07	0.07
Own child in house	0.45	0.40

ATUS, American Time Use Survey.

### Measures

As of 2021, 36 states and the District of Columbia have implemented either medical (MCL) or recreational cannabis laws (RCL). The time at which these laws became effective was determined by various sources, including McMichael et al. (2020),^17^ procon.org,^18^ pdaps.org,^19^ news articles, and Westlaw research on legal provisions. Table 2 displays the status of state medical and recreational laws, alongside their years of enactment. The primary measures of interest were coded as binary variables, indicating whether an MCL or RCL was in effect on a specific day in a given year for each state. Because each state implemented cannabis access laws at different times, both state- and year-fixed effects were included in the regression to control for unobserved heterogeneity across states and over time.

**Table 2:** Adoption of Medical and Recreational Marijuana Laws by Year

State	MCL year	RCL year
Alabama	2021	—
Alaska	1998	2015
Arizona	2010	2020
Arkansas	2016	—
California	1996	2016
Colorado	2000	2012
Connecticut	2012	2021
Delaware	2011	—
Washington, District Columbia	2011	2015
Florida	2017	—
Hawaii	2000	—
Illinois	2014	2020
Louisiana	2019	—
Maine	1999	2016
Maryland	2014	—
Massachusetts	2013	2016
Michigan	2008	2018
Minnesota	2014	—
Missouri	2018	—
Montana	2004	2021
Nevada	2000	2017
New Hampshire	2013	—
New Jersey	2010	2021
New Mexico	2007	2021
New York	2014	2021
North Dakota	2016	—
Ohio	2016	—
Oklahoma	2018	—
Oregon	1998	2015
Pennsylvania	2016	—
Rhode Island	2006	—
South Dakota	2021	—
Utah	2018	—
Vermont	2004	2018
Virginia	2020	2021
Washington	1998	2012
West Virginia	2019	—

MCL, medical cannabis law; RCL, recreational cannabis law.

The primary outcome of interest was average nightly sleep duration, measured in minutes. By arranging the ATUS data at the activity level rather than at the individual level, it becomes possible to examine activity start and stop times and test whether sleep changes induced by cannabis access laws affect the start and stop times for sleeping. Falling asleep time was coded to include start times between 7:30PM and 4:00AM. Waking up time was coded as stop times between 4:00AM and 10:00AM. To examine whether sleep changes are the result of individuals substituting sleep time for other activities, the main categories of time use in the ATUS were also independently regressed on the passage of RCLs. Recorded activities included naps, relaxing and leisure, education, eating and drinking, work, and social events. The nap variable was coded to record daytime sleep between the hours of 11:30AM and 7:30PM. The social events category was also analyzed as time spent alone, with a household or nonhousehold member, or at home.

In the main analysis, all respondents aged 18–65 years were included. In the secondary analysis, the sample was stratified by age and sex because existing first-stage literature generally suggests that cannabis use increases after RCL passage are most pronounced for males and for those aged >24 years.^20^ For each secondary regression, the subgroup of interest was omitted as a control. When running regressions for the effect of MCLs and RCLs on the sleep duration of those aged 18–24 years, age was excluded as a control variable. Demographic information, including age, sex, and race, was examined as covariates.

### Statistical Analysis

A two-way-fixed-effects difference-in-difference regression was estimated using the following specification:

$$Yi_{st}=\beta_{1}MCL_{st}+\beta_{2}RCL_{st}+\pi Xi_{st}+\gamma_{s}+\lambda_{t}+\varepsilon_{st},$$

where Y denotes minutes of sleep for individual i in state s at time t. MCL_st_ refers to whether state s had a MCL at time t, whereas RCL_st_ indicates whether state s had a RCL in place at time t. X is a vector of controls for whether the individual has kids aged <13 years in the household, age, sex, marital status, weekday indicator, holiday indicator, race, veteran status, indicator for whether a state’s average annual sunset time is earlier than 6:30PM, and employment status. γ represents state-fixed effects, and λ represents year-fixed effects. The coefficients of interest in this regression are β_1_ and β_2_, where β_1_ represents the average treatment effect of MCLs on sleep duration, indicating the change in minutes of sleep associated with the implementation of MCLs. β_2_ represents the average treatment effect of RCLs. Errors are clustered at the state level.

Analyses were conducted both including and excluding California to address concerns that California’s early and unique policy environment might confound estimates because California’s 1996 MCL was unusually broad and is often regarded as providing de facto recreational access. Event study models were also constructed to assess changes in average sleep duration before and after the implementation of MCLs and RCLs, ensuring that any observed changes in sleep are not driven by pre-existing trends or other confounding factors. All analyses were conducted in 2023.

## RESULTS

Table 3 shows the difference-in-differences estimates for the association between cannabis access laws and sleep duration. RCLs were associated with an average reduction in sleep of 5.37 minutes per night (99% CI=0.91, 9.83). Similar results were observed when California residents were excluded from the sample, with an associated reduction of 5.69 minutes per night (95% CI=10.49, 0.89). Although not statistically significant, MCLs were associated with an increase in average sleep duration by 1.79 minutes per night.

**Table 3:** Two-Way FE Difference-In-Differences Regression Results

	(1)	(2)	(3)
Variables	No controls	Full set of controls	Exclude California
			
MCL	1.87	1.79	1.38
	(1.61)	(1.57)	(1.52)
RCL	−4.44**	−5.37***	−5.69**
	(1.77)	(1.73)	(2.45)
Observations	175,493	175,493	157,990
Controls	No	Yes	Yes
State FE	Yes	Yes	Yes
Year FE	Yes	Yes	Yes
CA included	Yes	Yes	No

*Note:* Boldface indicates statistical significance (***p<0.01 and **p<0.05).

Robust SEs are in parentheses.

CA, California; FE, fixed effect; MCL, medical cannabis law; RCL, recreational cannabis law.

In examining differences in sleep by sex and age, the passage of RCLs was associated with an average of 6.2 minutes of sleep lost for males and 4.36 minutes for females. The relationship between RCLs and sleep is most significant for males and for those aged at least 25 years. The passage of MCLs was associated with an increase in sleep of 10.6 minutes per night (95% CI=0.80, 20.40) only for those aged 18–24 years. In all other specifications, the MCL coefficient is not statistically significant. Of each additional category of time use recorded, the only activity significantly affected by the passage of RCLs was eating and drinking, with an associated increase of 1.90 minutes (95% CI= 0.45, 3.35) (Table 4, Table 5). RCLs were associated with later sleep onset, with individuals observed to fall asleep an average of 7.14 minutes later at night (99% CI=3.12, 11.16), whereas MCLs were associated with earlier sleep onset of 3.31 minutes (95% CI=0.69, 5.93). There were no significant changes in waking-up times in response to either MCLs or RCLs (Table 6).

**Table 4:** Heterogeneous Effects, Sex, Age, and Mechanism Regression Results: Heterogeneous Effects, Sex, and Age

	(1)	(2)	(3)	(4)	(5)
Variables	Full sample	Male	Female	18–24	≥25
					
MCL	1.79	3.53	-0.06	10.60**	0.42
	(1.57)	(2.52)	(2.25)	(5.00)	(1.77)
RCL	−5.37***	−6.21*	**−**4.36	2.71	−6.06***
	(1.73)	(3.24)	(2.71)	(8.03)	(2.08)
Observations	175,493	79,002	96,491	13,907	161,586
Controls	Yes	Yes	Yes	Yes	Yes
State FE	Yes	Yes	Yes	Yes	Yes
Year FE	Yes	Yes	Yes	Yes	Yes

*Note:* Boldface indicates statistical significance (***p<0.01, **p<0.05, and *p<0.01).

Robust SEs are in parentheses.

FE, fixed effect; MCL, medical cannabis law; RCL, recreational cannabis law.

**Table 5:** Heterogeneous Effects, Sex, Age, and Mechanism Regression Results: Mechanism Regression Results

	(1)	(2)	(3)	(4)	(5)	(6)	(7)
Variables	Sleep	Naps	Relaxing and leisure	Education	Eating and drinking	Work	Social events
							
RCL	−5.37***	-0.64	−3.11	2.72	1.90**	1.18	−0.25
	(1.73)	(0.59)	(3.26)	(1.79)	(0.74)	(3.41)	(0.46)
Observations	175,493	175,493	175,493	175,493	175,493	175,493	175,493
Controls	Yes	Yes	Yes	Yes	Yes	Yes	Yes
State FE	Yes	Yes	Yes	Yes	Yes	Yes	Yes
Year FE	Yes	Yes	Yes	Yes	Yes	Yes	Yes

*Note:* Boldface indicates statistical significance (***p<0.01, **p<0.05, and *p<0.1).

Robust SEs are in parentheses.

FE, fixed effect; RCL, recreational cannabis law.

**Table 6:** Falling Asleep and Waking Up Times

	(1)	(2)
Variables	Falling asleep	Waking up
MCL	−3.31** (1.34)	0.25 (1.34)
RCL	7.14***	1.77
	(1.56)	(2.08)
Observations	177,495	170,193
R-squared	0.04	0.11
Controls	Yes	Yes
State FE	Yes	Yes
Year FE	Yes	Yes

*Note*: Boldface indicates statistical significance (***p<0.01 and **p<0.05).

Column 1 indicates when respondents start sleeping, whereas Column 2 refers to when respondents stop sleeping. Therefore, a positive coefficient in Column 1 corresponds to falling asleep later (sleeping less), whereas a positive coefficient in Column 2 would correspond to waking up later (sleeping more). Column 1 includes start times between 7:30PM and 4:00AM. Column 2 includes stop times between 4:00AM and 10:00AM.

FE, fixed effect; MCL, medical cannabis law; RCL, recreational cannabis law.

**Figure 2:**
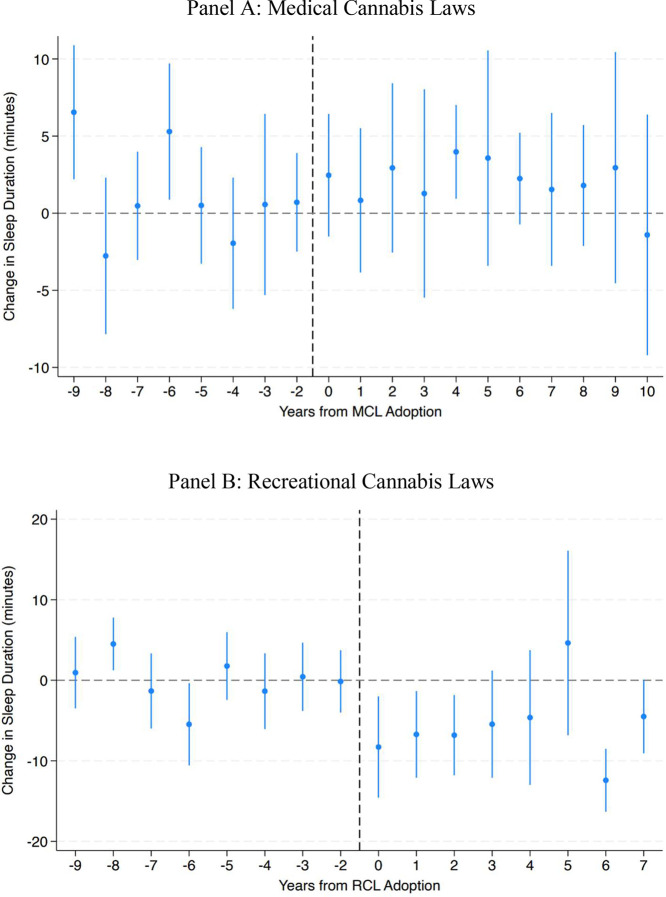
Event Studies for Medical and Recreational Cannabis Laws. (A) Medical cannabis laws. (B) Recreational cannabis laws.

Figure 2 provides the event studies for MCLs and RCLs, centered on years before and after treatment. These figures support the main regression results, indicating no change in sleep associated with the passage of MCLs and a reduction in sleep for that of RCLs. This decrease remains stable, and the lack of evidence for pretrends in these figures helps to alleviate concerns about parallel trends assumptions in the difference-in-differences design.

## DISCUSSION

This study finds that RCLs are associated with a reduction in average nightly sleep duration, particularly among males and those aged >24 years. These findings are consistent with existing literature, which suggests that cannabis use increases after recreational legalization are most pronounced among these groups.^20^ ATUS respondents reported a reduction in sleep duration after the passage of RCLs, with no change in the total number of minutes reported for all activities. However, the substitution from sleep to other activities is not driven by any singular activity but instead by multiple activities, such that the effects are too small to detect with the main time use categories of the ATUS. Furthermore, RCLs were not associated with an increase in socialization-related activities that take place with other people or that take place outside the home, suggesting that later sleep onset is not primarily driven by increased socialization.

The observed association between MCLs and increased sleep duration only among those aged 12–24 years is not well understood. One possible explanation for the lack of impact of MCLs on overall sleep duration is that insomnia or other sleep disorders are not qualifying conditions—medical conditions for which doctors can legally prescribe cannabis—in any state with an MCL in place. In contrast, RCLs permit broader access, allowing users to self-medicate for sleep disorders in states where RMLs are in place. Although both MCLs and RCLs are associated with changes in sleep timing at night, people do not seem to compensate for sleep changes by changing when they wake up. These findings are also consistent with prior studies suggesting that sleep changes occur more at night than in the morning owing to fixed work and school schedules and the fact that morning obligations are usually less flexible than nighttime commitments.^21^

Although the observed reduction in sleep is modest, it may still be meaningful at an individual level over time. Sleep deterioration causes reductions in earnings for workers,^22^ increases in fatal car crashes,^7^ and health consequences such as increased incidence of heart attacks.^23^ In a discrete choice experiment, Roy and colleagues^24^ (2015) found that those with insomnia were willing to pay an average of $66.69 per month for a treatment that led to an hour increase in nightly sleep. This suggests that individuals seeking sleep improvements may rationally turn to any available options, including the drug, but may not fully account for its costs. Cannabis users may also be staying up later at night to rationally enjoy the psychoactive high of the drug, and others may self-medicate by consuming cannabis for sleep therapy without recognizing its potential negative effects on sleep. Cannabis could also disrupt sleep patterns in ways that do not occur immediately. For example, although cannabis may initially reduce sleep latency onset, it can impair overall sleep quality and lead to disruptions the next day.^12^ These disruptions may compound over time, leading to irregular sleep schedules. This would also suggest caution for consumers who turn to cannabis as a sleep aid, medical practitioners who might recommend the drug for sleep improvements, and policymakers considering sleep problems as a qualifying condition for cannabis in MCL states.

Sleep duration and sleep latency onset are just 2 parts of sleep health. Another important aspect of sleep not explored in this paper is sleep quality, which is related to rapid-eye-movement sleep. Information on rapid eye movement is not available in the data used in this study. Moreover, cannabis use could impact sleep hygiene in other ways, such as triggering forgetfulness before bedtime or altering the regularity of one’s sleep schedule, although these aspects of sleep health are beyond the scope of this paper.

### Limitations

This study used a two-way-fixed-effects difference-in-differences model, which has known limitations. The comparison of later treated states with early treated states can produce wrong-signed and, thus, biased estimates under the traditional two-way-fixed-effects framework.^25^ Although event-study analyses presented in this study showed no evidence of pretrends, results should still be interpreted with caution.

This study relied on survey-based sleep data from ATUS rather than objective clinical measures, potentially introducing classical measurement errors and bias estimates toward the null. In addition, the data limit the ability to assess sleep quality nor can it determine whether reductions in sleep duration are offset by improved sleep quality. The ATUS also does not differentiate between time spent trying to fall asleep and time intentionally spent awake, potentially obscuring behavioral patterns related to cannabis use. Cannabis access laws were coded as a binary variable on the basis of their dates of passage, although legal variations between states such as dispensary access, enforcement, or qualifying conditions are not fully captured by the MCL and RCL variables. This study assumed that states without RCLs served as comparison groups but did not account for potential spillover effects from cross-border access to cannabis, which could lead to biased estimates toward the null or heterogeneous treatment effects that are not fully captured by the traditional two-way-fixed-effects framework. This study used cannabis access laws as a proxy for cannabis access and did not directly measure cannabis use or its mode of administration. In addition, this study did not capture differences in cannabis formulation, including compounds such as tetrahydrocannabinol, cannabidiol, and cannabinol, which have distinct pharmacological effects.^26^ RCLs often do not apply to products containing cannabidiol or cannabinol because they typically only mandate that tetrahydrocannabinol content is below certain thresholds. Owing to these variations, the findings of this study cannot be used to determine whether associations with sleep behavior are directly attributable to cannabis consumption. Finally, the large sample size may contribute to the detection of small effects that may not be meaningful at the individual level. Although the observed change in sleep duration after the passage of RCLs is statistically significant, its clinical and public health significance is less clear. This paper does not establish causality, but it contributes to the growing literature by estimating associations between cannabis access laws and sleep behavior. Future research should continue to explore how these laws interact with public health, particularly in sleep health and other behavioral outcomes.

## CONCLUSIONS

This paper provides new evidence of the associations between cannabis access laws and sleep. MCLs were not associated with significant changes in sleep duration, whereas RCLs were linked to later sleep onset and an estimated reduction of 38 minutes of sleep each week. This small nonclinically significant effect size also suggests that RCLs and MCLs do not differ in any meaningful way in terms of sleep and thus could be potentially viewed as a positive for proponents of recreational cannabis legalization. Beyond the individual effects of cannabis access laws on sleep, these results provide crucial implications for policymakers, healthcare providers, and consumers in shaping public health strategies and regulations for cannabis access.
